# Genomic Sequencing of Dengue Virus Strains Associated with Papua New Guinean Outbreaks in 2016 Reveals Endemic Circulation of DENV-1 and DENV-2

**DOI:** 10.4269/ajtmh.21-1292

**Published:** 2022-07-05

**Authors:** Marinjho Jonduo, Matthew J. Neave, Sarah Javati, Dorothy Abala, Eric Bilo, Anthony Kini, Janlyn Kumbu, Moses Laman, Leanne J. Robinson, Leo Makita, Melinda Susapu, William Pomat, Mohammad Yazid Abdad, David T. Williams, Paul F. Horwood

**Affiliations:** ^1^ Papua New Guinea Institute of Medical Research, Goroka, Eastern Highlands Province, Papua New Guinea;; ^2^ CSIRO Australian Centre for Disease Preparedness, Geelong, Victoria, Australia;; ^3^ Central Public Health Laboratory, Port Moresby, Papua New Guinea;; ^4^ Papua New Guinea Institute of Medical Research, Madang, Madang Province, Papua New Guinea;; ^5^ Burnet Institute, Melbourne, Victoria, Australia;; ^6^ Papua New Guinea National Department of Health, Port Moresby, Papua New Guinea;; ^7^ Centre for Tropical Medicine and Global Health, Nuffield Department of Medicine, University of Oxford, United Kingdom;; ^8^ Mahidol-Oxford Tropical Medicine Research Unit, Faculty of Tropical Medicine, Mahidol University, Thailand;; ^9^ James Cook University, College of Public Health, Medical and Veterinary Sciences, Townsville, Queensland, Australia

## Abstract

Over the past decade, the Pacific region has experienced many arboviral outbreaks, including dengue, chikungunya, and Zika viruses. Papua New Guinea (PNG) has a high burden of arboviral diseases, but there is a paucity of knowledge about the epidemiology and circulation of these viruses in the country. In this study, we report investigations into suspected arboviral outbreaks of febrile disease in PNG from December 2015 to June 2017. DENV-1 and DENV-2 were the mostly commonly detected viruses, and low circulation of DENV-3 and ZIKV was also detected. DENV-4 and CHIKV were not detected during this period. Full genome sequencing of selected positive samples revealed that circulation was dominated by endemic indigenous strains belonging to DENV-1 (genotype IV) and DENV-2 (genotype C) that have been present in the country for up to a decade. A DENV-2 sublineage was also identified that has been associated with outbreaks of severe dengue in both PNG and the Solomon Islands.

Dengue is an ongoing public health threat in many tropical countries, with > 100 million dengue infections and > 40,000 deaths per year.^1^ Dengue is primarily transmitted by *Aedes aegypti* and *Aedes albopictus* mosquitoes and is caused by four genetically and antigenically related types of dengue virus (DENV 1–4) belonging to the genus *Flavivirus* and family *Flaviviridae*. The clinical manifestations of dengue can range from asymptomatic infections, to mild-moderate febrile illness, through to severe dengue, which is a potentially fatal complication characterized by plasma leakage and hemorrhagic manifestations.

Papua New Guinea (PNG) is the largest Pacific Island nation, with a population of ∼9 million people. Health and social indicators in the country are poor compared with regional and global averages. Dengue circulation has been confirmed in PNG since 1944, when the DENV-2 type strain (DENV-2_New Guinea C_) was isolated from a U.S. soldier.^2^ Indeed DENV-1 was also isolated from soldiers stationed in PNG in the same year.^2^ In contrast to other settings in the south Pacific, where dengue circulation follows an epidemic–outbreak pattern, recent studies suggest that there is persistent circulation of endemic DENV strains in PNG.^3^^,^^4^ Although dengue outbreaks have been reported in PNG since the 1970s, severe dengue with hemorrhagic signs had not been reported in the country until an outbreak in Port Moresby (the PNG capital) in 2016.^5^ To gain a better understanding of DENV circulation in PNG and to investigate possible associations with severe dengue, we conducted full genome sequencing and phylogenetic analysis of DENV strains associated with outbreaks during December 2015–June 2017, including strains associated with severe dengue in Port Moresby in 2016.

Serum samples were collected from clinical cases between November 2015 to August 2017 as part of outbreak investigations conducted by the PNG National Department of Health. Outbreaks were identified after reports of clusters of acute febrile illnesses that were negative by malaria RDT testing. Serum samples were collected from patients and sent to the PNG Institute of Medical Research for testing. Nucleic acids were extracted from serum samples using the Qiagen DNeasy Blood and Tissue kit, according to the manufacturer’s instructions. Extracts were tested for DENV 1-4, chikungunya virus (CHIKV), and Zika virus (ZIKV) using previously published assays.^6^^–^^8^

Direct viral genome sequencing was attempted for all samples in which the target was detected with a sufficiently high viral load (CT value < 28). Nucleic acid extracts were sent to Macrogen (Seoul, South Korea) where library preparation was conducted using the Nextera XT kit (Illumina, Inc.) and next-generation sequencing was completed using the NovaSeq 6000 (Illumina, Inc.).

Human-derived sequences were first removed from the raw reads by mapping to the human reference genome, build 38 (NCBI Accession GCF_000001405.39), using the *bbduk.sh* command in bbmap v.38.84 (sourceforge.net/projects/bbmap). The nonhuman reads were then “cleaned” by removing Illumina adapters, trimming when the phred quality dropped below 20, and discarding remaining reads shorter than 50 bp with Trimmomatic v.0.38.^9^ The cleaned reads were then assembled using Trinity v.2.8.5^10^ with the default parameters. Assembled transcripts were annotated using diamond blastx v.0.9.24.125^11^ against the National Center for Biotechnology Information (NCBI) nr database, and transcripts matching to dengue virus were extracted for further cleaning and analysis. To ensure accurate assemblies, all cleaned reads were mapped to the dengue transcripts using Bowtie v.2.3.4,^12^ and the results checked for ambiguities in Geneious v.10.2.3.

The high-quality complete dengue genomes were then used in a phylogenetic analysis with backbone dengue sequences downloaded from NCBI’s GenBank database. All complete dengue genomes from NCBI containing a minimum set of meta-data were used for both DENV-1 and DENV-2 analyses using a modified Nextstrain workflow.^13^ The dengue repository from Nextstrain (https://github.com/nextstrain/dengue.git) was cloned using git and the config and meta-data files modified to include the NCBI genomes, plus those generated here. Snakemake v.5.26.1^14^ was used to run the workflow and the generated newick tree was drawn and annotated using GGTREE v.1.14.6.^15^ The zoomed portion of the trees was generated by extracting sequences of interest and aligning them with MAFFT v.7.407^16^ using the auto-flag to automatically select the most appropriate alignment parameters. Maximum likelihood phylogenetic trees were then generated with IQ-TREE v.2.0.6^17^ with 1,000 bootstrap replicates and model test enabled to automatically determine the most appropriate evolutionary model for each dataset.

During the study period, outbreak investigations were carried out in Kiunga (Western Province), Port Moresby (National Capital District), Daru (Western Province), Wabag (Enga Province), Nipa (Southern Highlands Province), Sailum (Morobe Province), Kundiawa (Chimbu Province), and Rabaul (East New Britain Province) (Table 1). Target viruses were detected during three outbreak investigations (Kiunga, Port Moresby, and Daru), and there was evidence for the circulation of multiple viruses in all three of these locations. During the periods when samples were collected, DENV-1 and DENV-2 were the main arboviral pathogens circulating in PNG. However, DENV-3 (*N* = 2) and ZIKV (*N* = 2) were also detected in samples collected from Western Province. Dengue patients had an average age of 22.3 years, and 41.2% of patients were female.

**Table 1:** Results from real-time reverse transcriptase polymerase chain reaction screening for selected arboviruses from febrile illness outbreak investigations in Papua New Guinea, 2015–2017

Location	Date collection range	No. of samples	DENV-1 (%)	DENV-2 (%)	DENV-3 (%)	DENV-4 (%)	ZIKV (%)	CHIKV (%)
Kiunga, Western Province	Dec. 2015–Feb. 2016	60	22 (37)	0	2 (3)	0	1 (2)	0
Port Moresby, National Capital District	Mar. 2016–Mar. 2017	138	5 (4)	22 (16)	0	0	0	0
Daru, Western Province	Dec. 2015–Jul. 2016	68	1 (1)	4 (6)	0	0	1 (1)	0
Wabag, Enga Province	Apr. 2016	24	0	0	0	0	0	0
Nipa, Southern Highlands Province	May. 2016	5	0	0	0	0	0	0
Sailum, Morobe Province	Jun. 2017	18	0	0	0	0	0	0
Kundiawa, Chimbu Province	Feb. 2016–Jun. 2016	6	0	0	0	0	0	0
Rabaul, East New Britain Province	Mar. 2016–Jun. 2017	7	0	0	0	0	0	0

CHIKV = chikungunya virus; DENV = dengue virus; ZIKV = Zika virus.

Full genome sequences were generated for three DENV-1 strains associated with an outbreak in Kiunga, Western Province (2016), and seven DENV-2 strains associated with outbreaks in Port Moresby (National Capital District, 2016) and Daru (Western Province, 2016). Sequence and phylogenetic analysis revealed that the PNG DENV-1 strains formed a monophyletic clade in Genotype IV (Figure 1, DENV-1 genome tree). The closest related viruses were previously reported from other locations in the Pacific, including PNG (2015-2016) and Micronesia (2004). The DENV-2 strains also formed a monophyletic clade in Genotype C and were closely related to viruses previously detected in PNG (2010-2016) and the Solomon Islands (2016) (Figure 2, DENV-2 genome tree).

**Figure 1.**
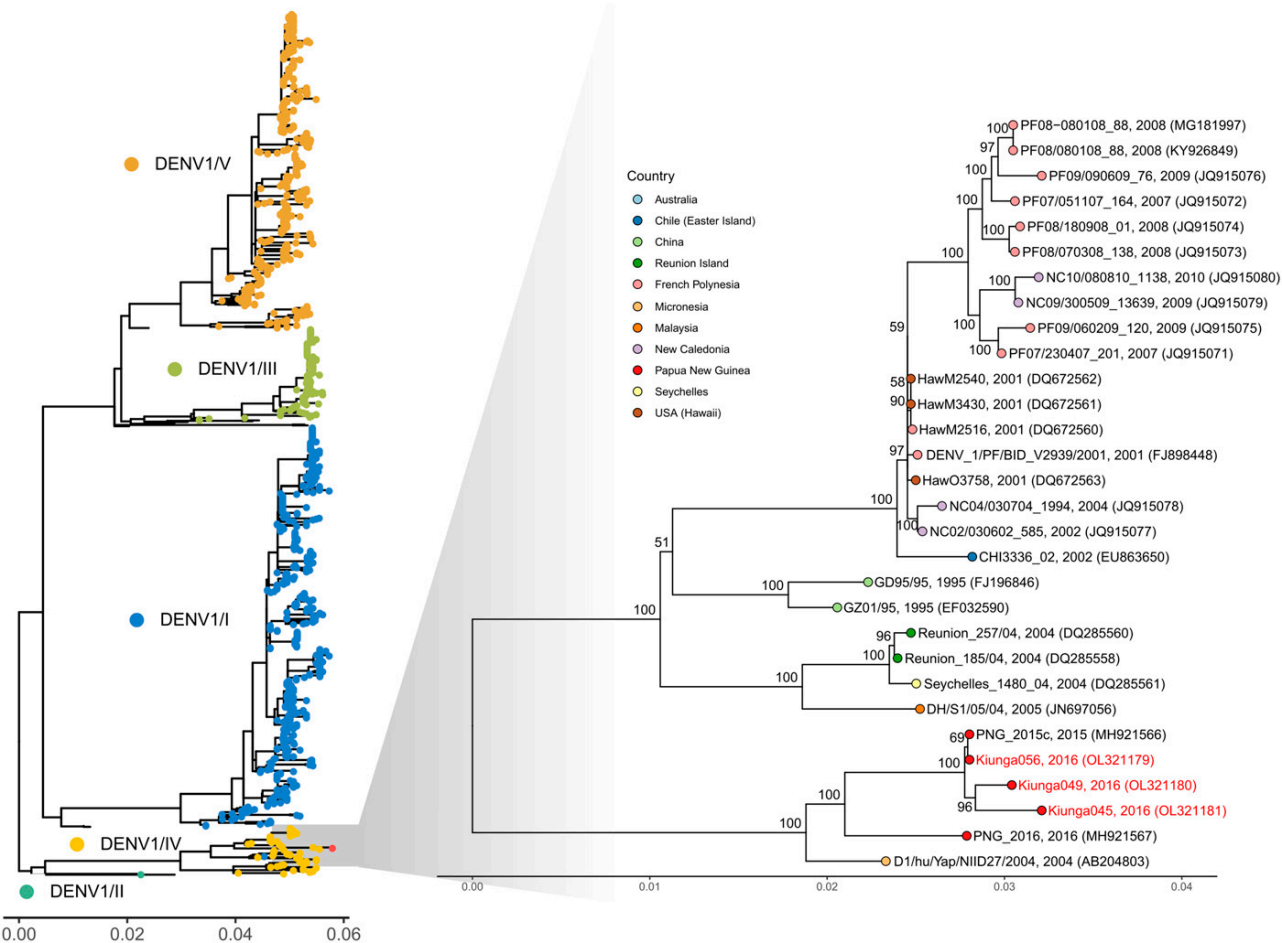
Maximum likelihood phylogenetic tree of whole DENV-1 genomes. The TIM2 model with empirical base frequencies and gamma rate heterogeneity was chosen as the best fit by IQ-TREE v.2.0.6. The percentage results from 1,000 bootstrap replicates are given on the nodes (if > 50%) and the scale represents the number of nucleotide substitutions per site. Sequences generated in this study are colored red.

**Figure 2.**
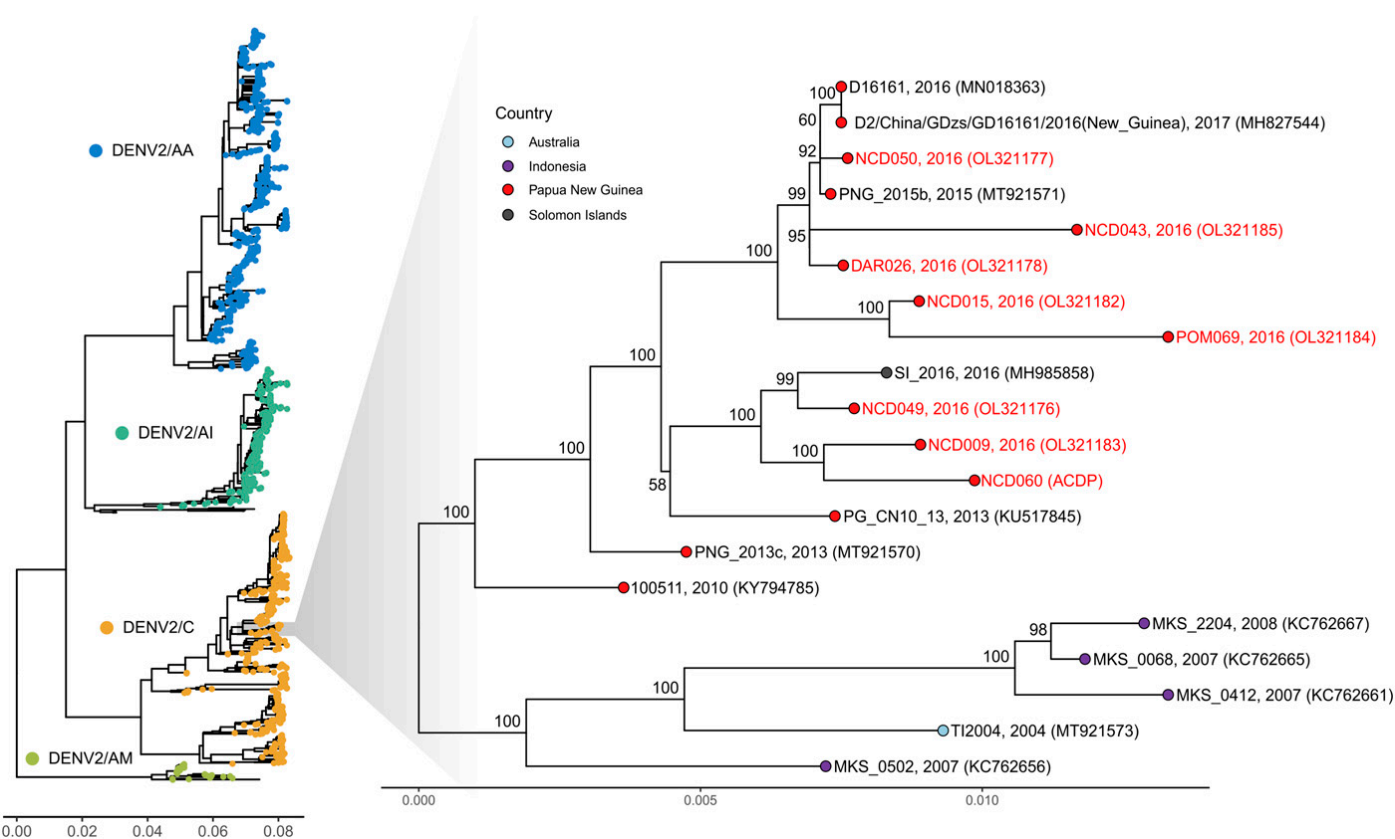
Maximum likelihood phylogenetic tree of whole DENV-2 genomes. The TN model with empirical base frequencies and gamma rate heterogeneity was chosen as the best fit by IQ-TREE v.2.0.6. The percentage results from 1,000 bootstrap replicates are given on the nodes (if > 50%) and the scale represents the number of nucleotide substitutions per site. Sequences generated in this study are colored red.

Envelope (E) glycoprotein gene phylogenetic trees were generated for the DENV-1 and DENV-2 strains to capture the majority of DENV sequences for which only the E-gene has been reported. Phylogenetic analysis of the E-gene for DENV-1 strains revealed that the viruses circulating in 2016 belonged to an endemic Genotype IV lineage that was first detected in the region (PNG, Fiji, Solomon Islands, northern Australia) in 2002–2003 (Supplemental Figure 1). Viruses belonging to this lineage have been detected in multiple countries throughout the Pacific region but have not been detected more widely. E-gene phylogenetic analysis of DENV-2 strains revealed that the 2016 isolates belonged to an endemic lineage of viruses that have circulated in PNG since at least 2010 (Supplemental Figure 2). This lineage likely evolved from a common ancestor of DENV-2 viruses originating from Indonesia, with viruses from Makassar (2007) basal to the PNG group. There is evidence that both of the DENV-1 and DENV-2 lineages endemic to PNG have spread to and caused outbreaks in other locations in the Pacific, such as the Solomon Islands, American Samoa, Fiji, Micronesia, and Australia.

In the current study, we found that DENV-1 and DENV-2 were the dominant arboviruses associated with febrile illness outbreaks in PNG during 2015–2017. Circulation of DENV-3 and ZIKV was also detected; however, they were not associated with outbreaks but rather co-circulation with DENV-1 and/or DENV-2. DENV-4 was not detected during the study, and indeed has been infrequently detected in PNG in the past. Interestingly, the only previous detection of DENV-4 strains from PNG were reported from two patients who traveled to Australia in July and October 2016,^3^ which corresponds to the same period as the present study. CHIKV was also not detected in this study, despite having caused a large nationwide outbreak during 2012–2013.^18^^,^^19^

Both the DENV-1 and DENV-2 viruses sequenced in this study belonged to endemic lineages that had been circulating in the country for up to a decade, confirming previous studies that have demonstrated hyperendemic circulation of indigenous DENVs in PNG. This study and other recent studies,^3^^,^^4^ suggest that new DENV strains are rarely introduced into PNG and circulation is dominated by homogenous groupings of viruses within each lineage. In 2016, a large outbreak of dengue was reported in Port Moresby that was associated with hemorrhagic manifestations.^5^ This is the first time that cases of severe dengue had ever been reported in PNG. Between August 2016 and April 2017, the Solomon Islands experienced its largest dengue outbreak on record with > 12,000 cases and 16 deaths (Figure 2, representative isolate from this outbreak: SI_2016, MH985858).^20^ In this study, we have confirmed that these outbreaks were caused by closely related strains of DENV-2, raising the question of whether viral evolution of the endemic PNG strain has led to the emergence of a more virulent lineage of this virus. Monitoring of the circulation of this sublineage is warranted to determine whether it is responsible for further outbreaks associated with severe cases.

Further research is needed to better understand the circulation of these DENVs in the PNG and determine the burden of disease associated with dengue infections. This is particularly pertinent as there is evidence to suggest that the PNG DENV lineages have been involved with outbreaks in other countries in the Pacific region.

## Supplemental files


Supplemental materials
