# Supplementary figures and images for: Genomic Sequencing of Dengue Virus Strains Associated with Papua New Guinean Outbreaks in 2016 Reveals Endemic Circulation of DENV-1 and DENV-2
Source: Am J Trop Med Hyg. 2022 Jul 5;107(6):1234–8. doi: 10.4269/ajtmh.21-1292 (PMC9768287; doi:10.4269/ajtmh.21-1292)

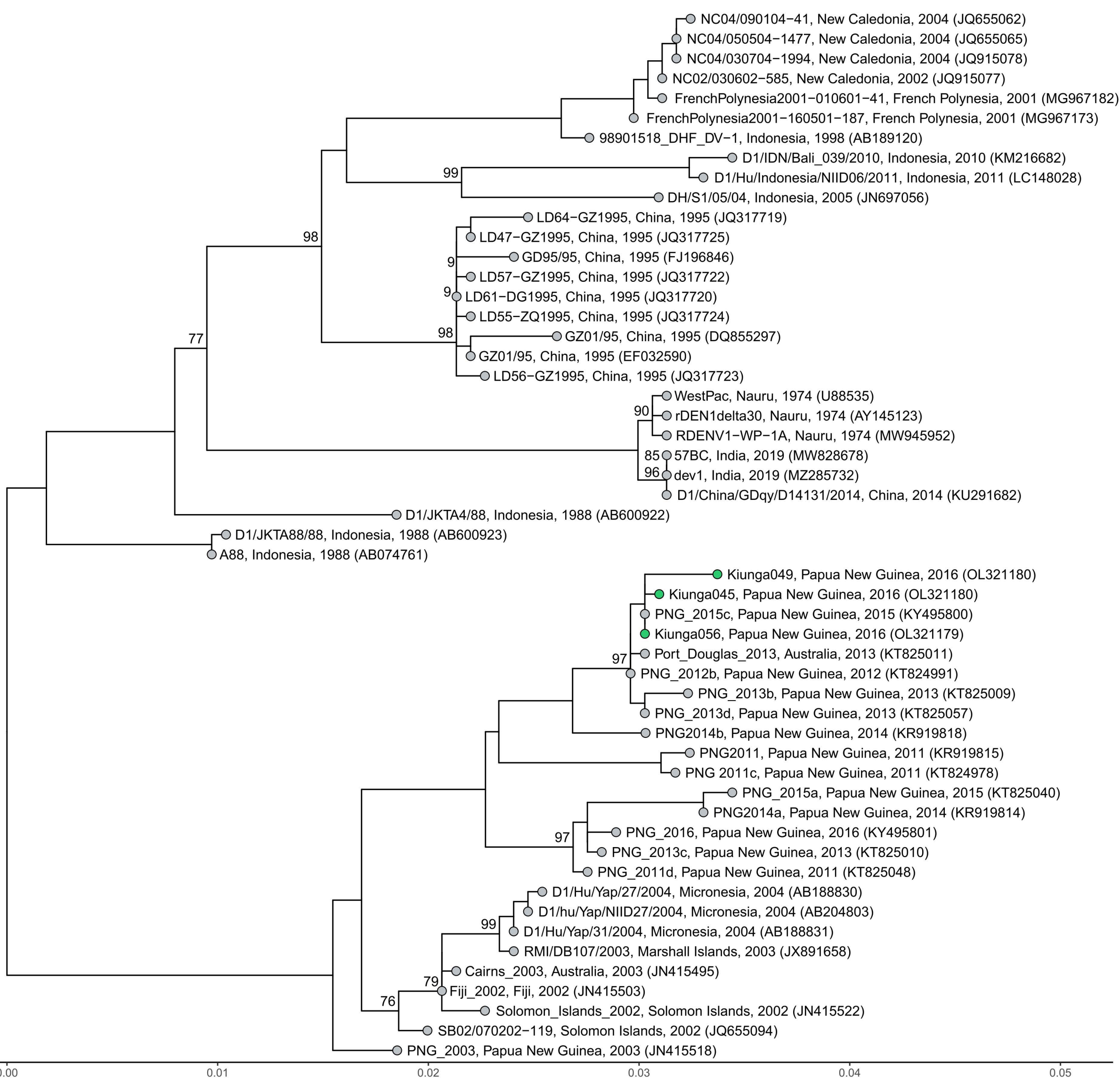

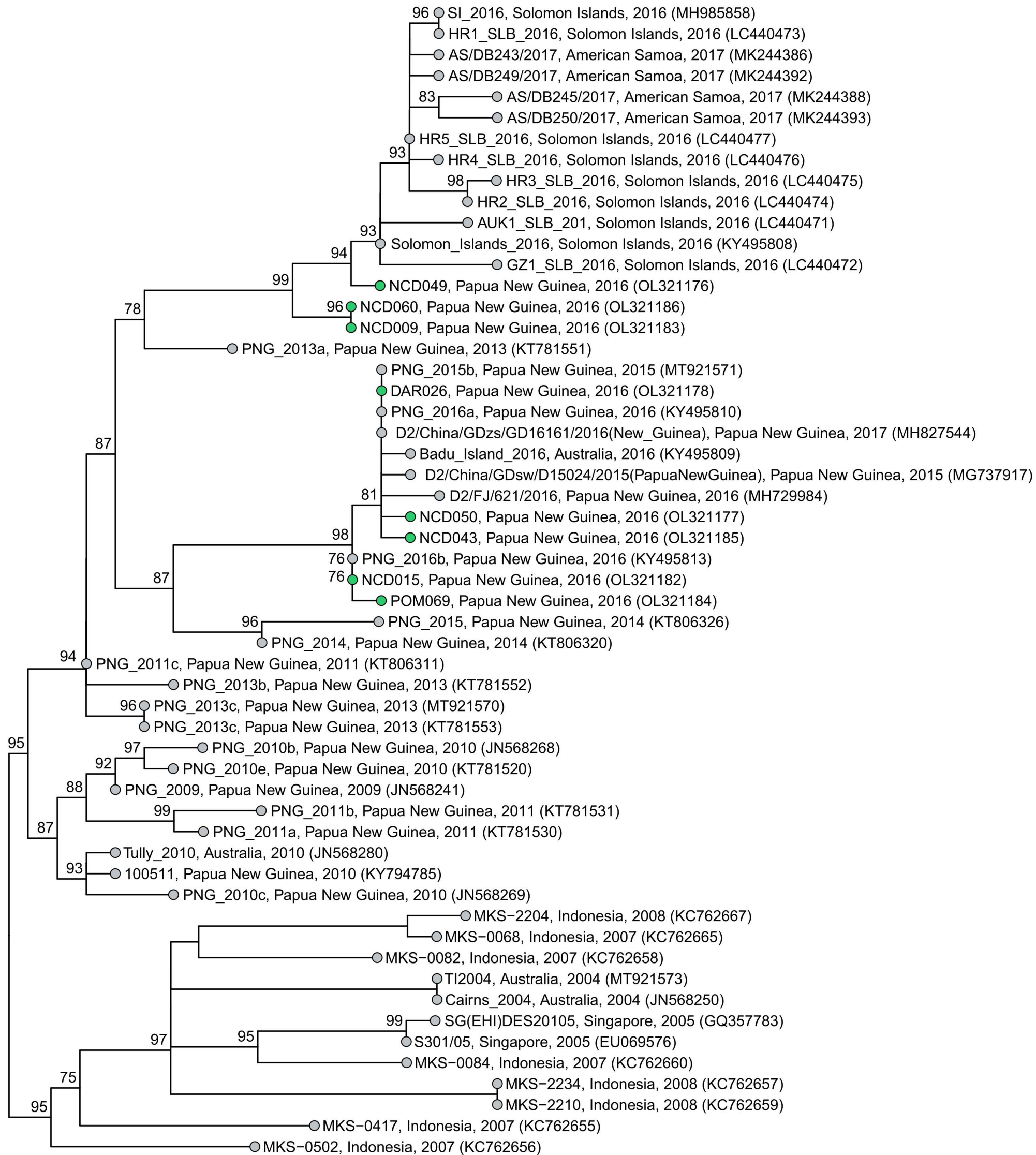

0.000 0.005 0.010 0.015 0.020

Supplement: Supplementary file 1 [file tpmd211292.SD1.pdf]
